# Influence of deposition technique on the structural and optical properties of CuS thin films for hole transport layers

**DOI:** 10.1038/s41598-025-13226-6

**Published:** 2025-09-30

**Authors:** Tushar A. Limbani, A. Mahesh, Shivani R. Bharucha

**Affiliations:** 1 C. L. Patel Institute of Studies and Research in Renewable Energy, Charutar Vidya Mandal University, New Vallabh Vidyanagar, Anand, 388121 Gujarat India; 2 N. V. Patel College of Pure & Applied Science, Charutar Vidya Mandal University, Vallabh Vidyanagar, Anand, 388120 Gujarat India

**Keywords:** Thin films, Energy levels, Structural characterization, Optical bandgap analysis, Perovskite solar cells, Low-cost fabrication methods., Materials science, Nanoscience and technology, Physics

## Abstract

This study presents a direct comparison of copper (II) sulfide (CuS) thin films fabricated by spin coating and doctor blade coating as hole transport layers (HTLs) in perovskite solar cells (PSCs). Both sets of films were fully characterized using Xray diffraction (XRD) to determine crystallinity and grain size, scanning electron microscopy (SEM) for morphology, energydispersive Xray spectroscopy (EDAX) for composition, ultraviolet–visible (UV–Vis) spectroscopy for optical bandgap, Xray photoelectron spectroscopy (XPS) for surface chemistry, and ultraviolet photoelectron spectroscopy (UPS) for energy-level alignment and found to be crystalline and phase‐pure. Doctor bladecoated films exhibited larger crystallite sizes (≈ 44.9 nm vs. 37.45 nm), slightly lower band gaps (3.15 eV vs. 3.23 eV), and despite higher microstrain and dislocation density, a closer valence band maximum (VBM = − 5.44 eV vs. − 5.99 eV) and conduction band minimum (CBM = − 2.29 eV vs. − 2.76 eV) to the MAPbBr₃ perovskite absorber. When these experimental parameters were incorporated into three‐dimensional drift–diffusion simulations under AM1.5G illumination, the doctor blade HTL yielded a projected power conversion efficiency of 41.7% driven by high charge density (> 10²⁴ cm⁻³) and low recombination rates (~ 1 × 10⁻⁵ s⁻¹) compared to 39.5% for the spincoated film. These results demonstrate that, while both deposition methods produce viable CuS HTLs, the doctor blade technique offers superior energylevel compatibility, scalable processing, and costeffectiveness for highefficiency, largearea PSC manufacturing.

**Supplementary Information:**

The online version contains supplementary material available at 10.1038/s41598-025-13226-6.

## Introduction

Perovskite-based solar cells (PSCs) have emerged as an up-and-coming next-generation photovoltaic technology owing to their excellent optoelectronic features, including elevated absorption coefficients, adjustable bandgaps, and outstanding charge transport capabilities^1,2^. These characteristics have resulted in quick progress in the efficiency of PSCs, exceeding 25% in a very short period^3^. The choice of appropriate electron transport layers (ETLs) and hole transport layers (HTLs) is crucial for the overall efficiency and stability of the devices^4^. The processes of thin-film deposition for functional layers, including spin coating and doctor blade coating, substantially affect the material characteristics, morphology, and performance of the resultant devices^5^. Although spin coating is prevalent for its ease, consistent film creation, and thickness control, doctor blade coating has lately garnered interest as a feasible alternative, especially for large-scale production^6^.

Spin coating and Doctor Blade coating are two essential methods in the manufacture of thin films for perovskite solar cells (PSCs), each presenting distinct benefits and problems according to the intended application and manufacturing scale^7^. Spin coating, a fundamental technique in laboratory research, is renowned for its accuracy and capacity to produce smooth, uniform thin films with regulated thickness and crystallinity^8^. These characteristics are essential for improving charge transfer and minimizing recombination losses, therefore optimizing device performance^9^. Nevertheless, the actual limits of spin coating, specifically considerable material waste, restrictions on substrate dimensions, and its unsuitability for large-area processing, present substantial obstacles to commercial scaling^10^.

The Doctor Blade coating technology has evolved as a revolutionary method, especially appropriate for large-scale and roll-to-roll production^11^. This technique integrates operational efficiency with minimized material waste and is proficient at applying uniform thin films over large surfaces^12^. Utilizing a blade to apply the liquid precursor solution, followed by regulated drying, Doctor Blade coating has shown significant promise to generate films with optoelectronic qualities like those obtained by spin coating^13^. Recent advancements in Doctor Blade technology underscore its potential for innovation. Research indicates that the optimization of coating parameters, specifically precursor concentration, blade speed, and drying conditions, can rectify slight variations in surface morphology and crystallinity, attaining performance metrics comparable to those of spin-coated films^14^. This versatility makes Doctor Blade coating a feasible and sustainable solution for scale PSC manufacture. Furthermore, its compatibility with upcoming flexible substrates and its integration with sustainable manufacturing purposes highlight its significance in the shift from laboratory to industrial production^15^. Although spin coating is a standard for accuracy and material homogeneity in research environments, the adaptability and scalability of Doctor Blade coating represent a significant change in the economical, high-throughput production of PSCs. Coordinating these strategies to use their distinct advantages may facilitate the development of new device designs and enhance the commercialization of perovskite technology^5^.

Even though there have been improvements, we still don’t fully understand how different coating methods affect the electronic, optical, and structural features of HTLs such as CuS. CuS, characterized by its inherent stability, p-type conductivity, and advantageous energy level alignment, is a good choice for HTL. Nonetheless, the influence of deposition-induced changes in crystallinity, morphology, and band alignment on the performance of CuS in perovskite solar cells remains inadequately investigated. Rectifying this deficiency is crucial for enhancing CuS films for effective and scalable PSC production^16^. These characteristics facilitate effective hole extraction and conveyance, which are essential for improving device performance^17^. The deposition technique is crucial in determining the structural, morphological, and optoelectronic characteristics of CuS films, influencing their efficacy as HTLs^18^. Methods such as spin coating and doctor blade coating have been used to produce CuS thin films, with each technique affecting factors such as crystallite size, microstrain, dislocation density, and surface smoothness. The deposition-driven attributes are essential for enhancing CuS in perovskite solar cells, providing increased efficiency and scalability.

This study presents a comprehensive comparative analysis of CuS thin films deposited via Doctor Blade and Spin Coating techniques, aiming to evaluate their suitability as HTLs in PSCs. Structural, morphological, optical, and electronic properties of the films were systematically characterized using XRD, SEM, UV-Vis spectroscopy, XPS, and UPS. These analyses enabled precise determination of crystallinity, surface features, optical bandgaps, work function, valence band edge, and band alignment with the MAPbBr₃ perovskite layer.

This study presents several novel contributions to the field of PSCs: (i) A direct, side-by-side comparison of Doctor Blade and Spin Coating deposition techniques for CuS-based HTLs, evaluated under identical processing and characterization conditions, ensuring a reliable performance comparison; (ii) A detailed investigation of interfacial energy level alignment between CuS and MAPbBr₃ perovskite, which is essential for understanding hole extraction dynamics and minimizing recombination losses, yet often underexplored in existing literature; (iii) The incorporation of experimentally derived parameters into 3D drift-diffusion simulations, enabling the isolation and quantification of HTL contributions to overall device behavior.

By combining comprehensive material characterization with device-level modeling, this study provides a unique, application-focused perspective on scalable HTL integration. The findings particularly emphasize the viability of Doctor Blade coating as a cost-effective, large-area deposition strategy for next-generation PSC manufacturing.

## Characterization

The characterization of the spin-coated and doctor blade-coated CuS films included a thorough examination of their structural, morphological, and optical characteristics using several sophisticated methods. EDAX measurements were performed using a system connected to a field emission scanning electron microscope (FEG-SEM, model XL-30). The XRD patterns of both films were obtained using a Bruker D8 Advance diffractometer with a Cu Kα radiation source (λ = 1.54060 Å). High-resolution pictures were acquired from the SEM study conducted using a FEG-SEM XL-30. The optical absorption spectra were obtained using a Perkin Elmer Lambda 19 spectrometer, spanning a wavelength range of 200 nm to 800 nm. Photoluminescence (PL) spectra were observed using “Microscope Photo Luminescence Spectrometer-flex One”. X-ray photoelectron spectroscopy (XPS) and ultraviolet photoelectron spectroscopy (UPS) were performed using a Thermo-Scientific NEXSA spectrometer. With an Al K-Alpha source (1486.6 eV), this device functioned inside the ultra-high vacuum region of 10^−8^ to 10^−10^ mbar with C 1 s as a reference.

## Experimental procedures

### Chemicals

Copper (II) sulfide pentahydrate (CuSO₄·5 H₂O) (≥ 99.99%, Sigma-Aldrich, ACS reagent grade) served as the principal component for the CuS precursor solution. Hydrochloric Acid (HCl) (37%, Merck, analytical grade) and Sodium Thiosulfate (Na₂S₂O₃) (≥ 99.0%, Sigma-Aldrich, ACS reagent grade) were used in the formulation of the precursor solutions. Ethanol (≥ 99.8%, Sigma-Aldrich, HPLC grade) and Acetone (≥ 99.5%, Sigma-Aldrich, analytical grade) were used for substrate cleaning. All compounds were used as obtained, without further purification. High-purity deionized (DI) water was used for cleaning and solution formulation throughout the studies.

### Preparation of CuS precursor solution

Initially, 0.4 g of copper (II) sulfide pentahydrate (CuSO₄·5 H₂O) was dissolved in 10 mL of deionized water to form an aqueous solution. Subsequently, 0.5 g of sodium thiosulfate (Na₂S₂O₃) was included in the solution as a reducing agent. The solution was acidified by the addition of 1 mL of strong HCl. The solution was agitated at 70 °C for 30 min until a homogenous mixture was obtained. The precursor solution was then chilled to ambient temperature before coating application.

### Doctor blade coating of CuS

**Fig. 1:**
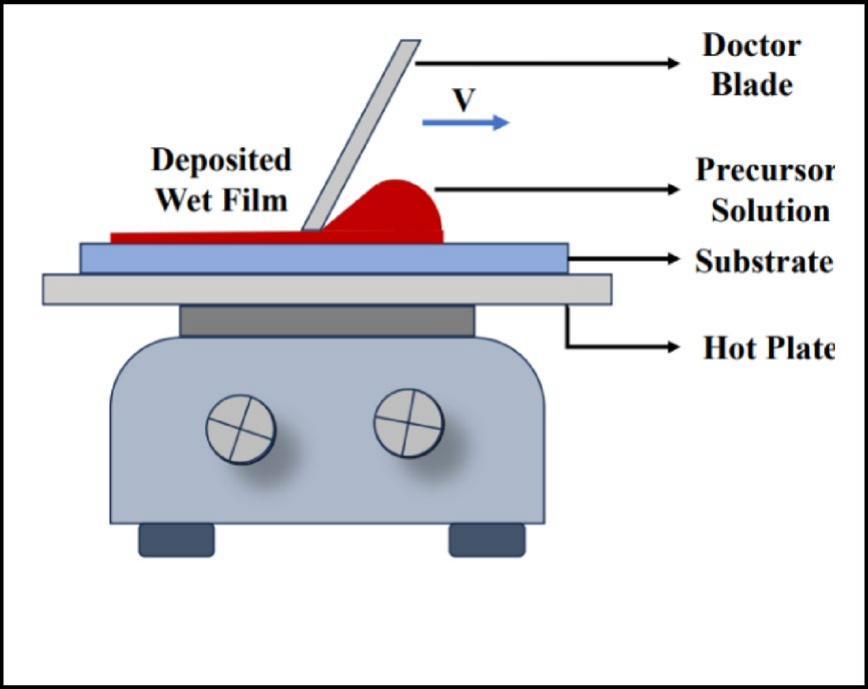
Doctor blade coating setup.

The CuS films were deposited with a comparable Doctor Blade process. The CuS precursor solution was applied to the glass substrate and evenly distributed using a doctor’s blade (See Fig. 1). The coating parameters, including blade speed (~ 50 mm/s) and gap height, were modified to provide a uniform and smooth layer. After coating, the substrate underwent annealing at 120 °C for 5 min to facilitate film formation and crystallization of CuS.

### Spin coating of CuS

The CuS films were prepared with the spin coating method. The CuS precursor solution was deposited in the center of the glass substrate, which was then spun at 1000 RPM for 10 s to uniformly distribute the solution across the surface. The procedure was executed twice to guarantee consistent coverage. After deposition, the substrate underwent annealing at 120 °C for 5 min to facilitate film formation and the crystallization of CuS.

## Results and discussion

**Fig. 2:**
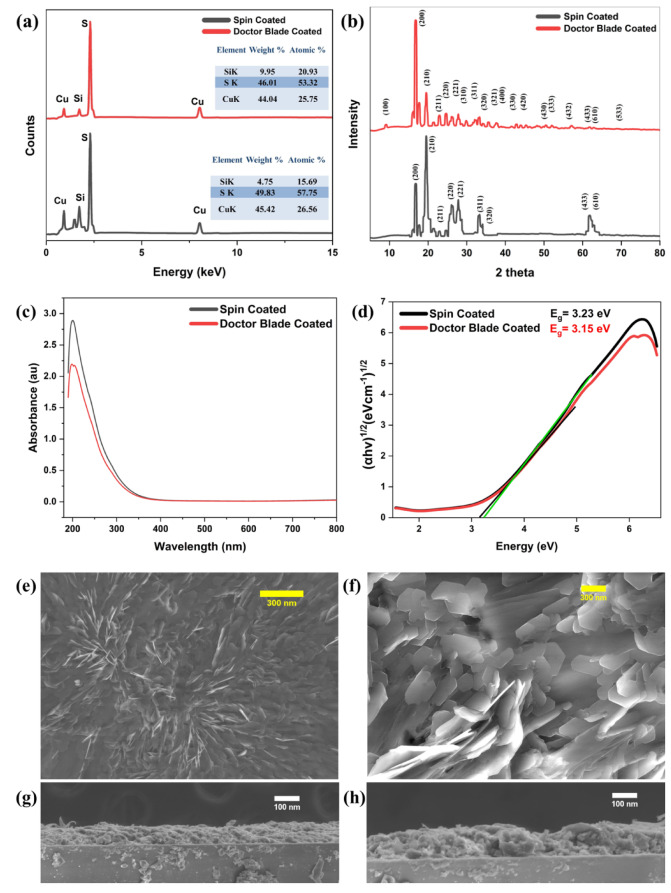
For both Spin- and Doctor Blade-coated thin film samples **(a)** EDAX analysis; **(b)** XRD patterns; **(c)** UV-Vis absorbance spectra; and **(d)** Tauc plots. **(e)** SEM image of the surface morphology of the Spin-coated film; **(f)** SEM image of the surface morphology of the Doctor Blade-coated film; **(g)** Cross-sectional SEM image of the Spin-coated film; **(h)** Cross-sectional SEM image of the Doctor Blade-coated film.

Figure 2a presents EDAX (energy dispersive X-ray analysis) spectra of CuS films prepared by spin-coating and doctor blade techniques. The EDAX spectra of the thin spin-coated CuS film exhibit well-defined peaks for copper (Cu), sulfur (S), and silicon (Si). The atomic percentage of Cu and S are 45.42% and 49.83%, respectively, indicating a uniform distribution of CuS. The EDAX spectra of the Doctor Blade-coated CuS film exhibit comparable peaks for Cu, S, and Si, with atomic percentages of 44.04% for Cu and 53.32% for S. The sulfur concentration is somewhat more than that of the Spin-coated film; nevertheless, this discrepancy is insignificant for the total composition for HTL applications. The Doctor Blade coating continues to provide a balanced and appropriate blend of CuS.

The silicon (Si) peak seen in both spectra is ascribed to the glass substrate, which comprises silicon dioxide (SiO₂). Due to the thinness of the CuS thin films, the electron beam used in EDAX might partly interact with the substrate, leading to observable silicon signals. The anticipated Si input from the substrate has not affected the compositional integrity or electronic properties of the CuS films. The relative intensities and elemental ratios correspond with the anticipated stoichiometry for CuS thin films. Spin-coated and doctor-blade-coated CuS films exhibited well-balanced compositions. The little variation in sulfur concentration does not substantially influence the material’s appropriateness for HTL in perovskite solar cells. Consequently, all procedures are essentially equal regarding elemental composition.

XRD data (Fig. 2b) indicate that the structural study of spin-coated and doctor-blade-coated CuS thin films demonstrates notable changes in their crystallographic characteristics, which affect their efficacy as hole transport layers in perovskite solar cells. The XRD examination, including the peak determination, crystallite size, strain, and dislocation density, was conducted according to the approach detailed in our previous investigations^19,20^.

The spin-coated CuS thin film displays a hexagonal structure (Crystallography Open Database (COD) CIF No. 9008368, P6_3/mmc space group), characterized by a d-spacing of 2.6758 Å, lattice parameters a = b = 3.796 Å and c = 16.38 Å, and an average crystallite size of 37.45 nm (Table 1). Figure 2b illustrates that the significant diffraction peaks at 2θ at 16.61°, 19.62°, 22.92°, 26.08°, 27.68°, 32.80°, 34.14°, 61.72°, and 62.92° locations correspond to higher-order planes, namely (200), (210), (211), (220), (221), (311), (320), (433), and (610), hence affirming a refined crystalline structure. The Spin-coated film exhibits a reduced microstrain (1.31 × 10^−3^) and a markedly decreased dislocation density (3.83 × 10^−4^ nm^−2^), signifying fewer crystal defects and enhanced crystallinity. This improved crystal structure enhances effective charge transmission in HTL applications.

**Table 1:** Structural data of CuS thin films coated using spin and Doctor blade techniques, derived from XRD measurements.

	d-Spacing (Å)	a = b (Å)	C (Å)	Crystallite size (nm)	Micro strain (ε)	Dislocation density (δ) (nm^−2^)
Spin coated	2.6758	3.796	16.38	37.45	1.31 × 10^−3^	3.83 × 10^−4^
Doctor blade coated	2.1463	3.796	16.38	44.9	2.67 × 10^−3^	6.89 × 10^−3^

Conversely, the doctor blade-coated CuS thin film exhibits a hexagonal structure (Crystallography Open Database (COD) CIF No. 9008368, P6_3/mmc space group) with marginally different properties. The d-spacing is consistent at 2.1463 Å, with lattice parameters maintained at a = b = 3.796 Å and c = 16.38 Å (Table 1). The crystallite size is comparatively greater at 44.9 nm, indicating enhanced grain growth. Figure 2b illustrates that the principal diffraction peaks align at 10.01°, 16.61°, 19.62°, 22.92°, 26.08°, 27.68°, 29.27°, 32.80°, 34.14°, 35.40°, 38.70°, 43.07°, 44.13°, 45.40°, 50.40°, 56.25°, 61.70°, 63.30°, and 69.26° corresponding to the crystal planes (100), (200), (210), (211), (220), (221), (310), (311), (320), (321), (330), (333), (400), (420), (430), (432), (433), (533), and (610). Nonetheless, the doctor blade-coated film exhibits a greater microstrain (2.67 × 10^−3^) and elevated dislocation density (6.89 × 10^−3^ nm^−2^), indicating a minor increase in crystal defects relative to the spin-coated film.

Significant variations in the relative intensities of the (200) and (210) peaks are observed between the two films. The spin-coated CuS film exhibits a higher intensity (210) peak compared to the (200) peak, indicating a preferred orientation along the (210) plane, attributed to uniform film formation and centrifugal forces during the spin coating process. This orientation may indicate a localised reordering or pseudo-cubic symmetry; however, the overall structure remains hexagonal, as verified by other consistent peak positions.

The structural differences between spin-coated and doctor blade-coated CuS films stem from their distinct deposition dynamics, film thicknesses, and solvent drying rates^21^. In the doctor blade process, the precursor solution dries more gradually, allowing crystals to grow larger (average size 44.9 nm compared to 37.5 nm), which reduces grainboundary scattering and enhances hole mobility. However, the same mechanical shearing action of the blade and non-uniform drying front introduce higher microstrain (2.67 × 10⁻³ vs. 1.31 × 10⁻³) and dislocation density (6.89 × 10⁻³ nm⁻² vs. 3.83 × 10⁻⁴ nm⁻²). While increased defect densities can act as charge-recombination centers, our UPS measurements and drift–diffusion simulations confirm that the improved energy‐level alignment with MAPbBr₃ and larger crystallites outweigh these drawbacks, resulting in net gains in hole extraction efficiency and predicted device performance.

Determining the optical bandgap of spin- and doctor blade-coated CuS films is essential to assess their suitability as HTLs in PSCs. A suitable bandgap ensures that the HTL remains transparent to visible light while enabling optimal energy-level alignment with the perovskite absorber layer. This facilitates efficient hole extraction and suppresses recombination losses at the interface. Comparing the bandgap values also helps identify the deposition method that best supports scalable and high-performance PSC architectures. The UV–Vis spectra (Fig. 2c) of the spin-coated CuS film reveal a sharp absorption onset at approximately 3.23 eV, with virtually no absorption in the visible range. This pronounced UV absorption edge confirms the film’s optical quality and guarantees maximal light transmission to the perovskite absorber. Such transparency, combined with the HTL’s favorable energy-level alignment, enhances overall device performance by improving light harvesting and charge extraction. The practical relevance of CuS in this architecture stems from its role as a hole transport layer rather than an absorber. Its wide optical bandgap renders it highly transparent throughout the visible spectrum, enabling maximal light transmission to the underlying perovskite absorber. Moreover, the distinct absorption edge and optimal energy‐level alignment with MAPbBr₃ facilitate rapid hole extraction and minimize recombination losses, thereby enhancing overall device performance. The UV-VIS spectrum (Fig. 2c) of the doctor blade-coated CuS film exhibits comparable absorption properties but with a little reduced peak intensity in the UV region. This decrease is negligible and does not significantly affect the film’s performance. The Tauc plot for the spin-coated CuS film (Fig. 2d) indicates a straight band gap of around 3.23 eV, advantageous for effective hole transport in HTL applications. The Tauc plot for the doctor blade-coated CuS film indicates a band gap of 3.15 eV, which is somewhat reduced relative to the spin-coated film. The 0.08 eV disparity in the bandgap between spin-coated and doctor blade-coated CuS films is determined using the Tauc plot method. Fluctuations in bandgap may also stem from the microstructural characteristics, surface morphology, and crystalline arrangement of the films, which influence the electrical structure of the materials.

The SEM picture (Fig. 2e) of the spin-coated CuS film displays a smooth and uniform surface characterized by well-defined crystallites. The shape is uniform and crucial for facilitating optimum charge transfer and minimizing recombination in the HTL. The SEM picture (Fig. 2f) of the doctor blade-coated CuS film exhibits a coarser and somewhat irregular surface in comparison to the spin-coated film. The cross-sectional SEM picture (Fig. 2g) of the spin-coated CuS film indicates an average thickness of around 90 nm. The slender and well-structured film facilitates effective charge transfer characteristics for the HTL. The cross-sectional SEM picture (Fig. 2h) of the doctor blade-coated CuS film reveals a layer with an average thickness of around 120 nm. The augmented thickness does not lead to any substantial deterioration of the film’s quality, and the additional thickness may even enhance hole transit efficiency in some instances. The increase in thickness of the doctor blade-coated film is minimal and does not substantially impact the overall performance of the HTL layer. Both techniques provide films with suitable thicknesses for efficient charge transmission.

Photoluminescence (PL) spectroscopy was employed to evaluate the optical and electronic quality of CuS thin films. Figure S1 presents the PL spectra of Spin Coated and Doctor Blade Coated films, with emission peaks observed at 434.3655 nm and 434.1254 nm, respectively. A detailed discussion is provided in the Supplementary Information.

**Fig. 3:**
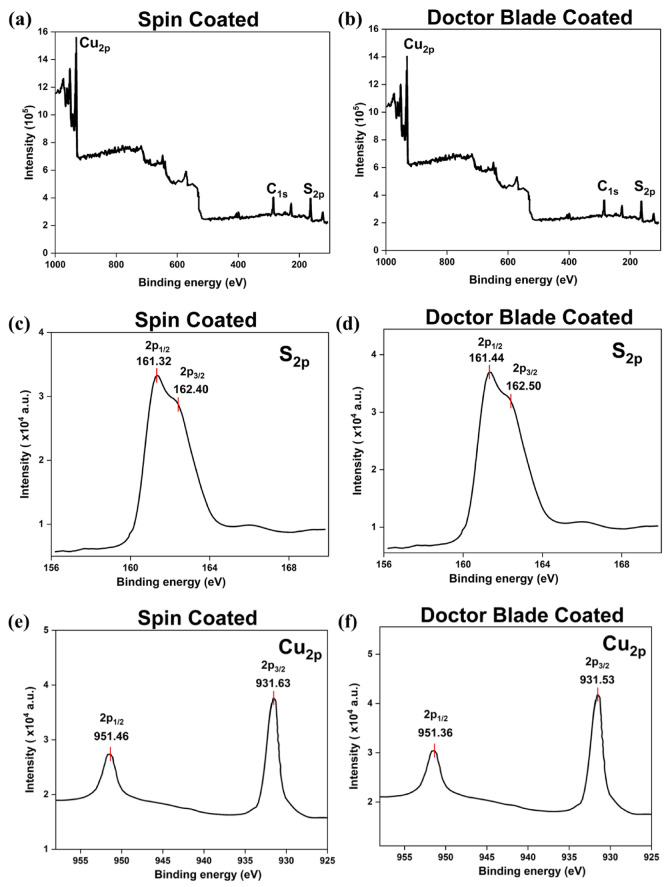
Comprehensive XPS scan of **(a)** Spin-coated CuS thin film; and **(b)** Doctor Blade-coated CuS thin film. Detailed S 2p spectrum for **(c)** Spin-coated CuS thin film; and **(d)** Doctor Blade-coated CuS thin film. High-resolution Cu 2p spectrum for **(e)** Spin-coated CuS thin film; and **(f)** Doctor Blade-coated CuS thin film.

Figures 2b and 3a show the XPS survey scan of CuS thin films using spin-coating and doctor blade coating methods. The survey spectra validate the existence of Cu 2p, S 2p, and a tiny C 1 s signal, indicating the effective deposition of CuS films. The C 1 s peak is attributed to using C 1 s as a reference for binding energy calibration; due to its surface sensitivity, substrate signals like Si were not detected. Figures 2d and 3c provide high-resolution scans of the S 2p core level for spin-coated and doctor blade-coated films, respectively. The Spin-Coated film (Fig. 3c) displays peaks at 161.32 eV and 162.40 eV, corresponding to the same S 2p states. Likewise, the peaks at 161.44 eV and 162.50 eV in the Doctor Blade-Coated film (Fig. 3d) correspond to the S 2p_1/2_ and S 2p_3/2_ states, indicative of sulfur in the S²⁻ oxidation state. The little discrepancy in binding energy (0.1 eV) and peak intensities between the two methods indicates nuanced variations in the sulfur environment or distribution inside the CuS films^22,23^. Figures 2f and 3e show the high-resolution scans of the Cu 2p core level for the spin-coated and doctor blade-coated films, respectively. The spin-coated film (Fig. 3e) exhibits Cu 2p_3/2_ and Cu 2p_1/2_ peaks at 931.63 eV and 951.46 eV, respectively. Likewise, the Cu 2p_3/2_ peak at 931.53 eV and the Cu 2p_1/2_ peak at 951.36 eV are seen for the doctor blade-coated film (Fig. 3f). The binding energies are indicative of copper in the Cu⁺ oxidation state, hence supporting the establishment of Cu-S bonds in the CuS phase. The lack of shake-up subsidiary peaks signifies the absence of Cu²⁺, suggesting no copper oxide contaminants. The negligible shift (0.1 eV) and little discrepancy in peak intensities between the two approaches indicate minimal changes in copper content or bonding environment inside the films. The XPS data indicate that the binding energies are almost equal, with slight changes in peak intensities and shifts suggesting differences in elemental distribution or film uniformity between the spin-coated and doctor blade-coated samples.

**Fig. 4:**
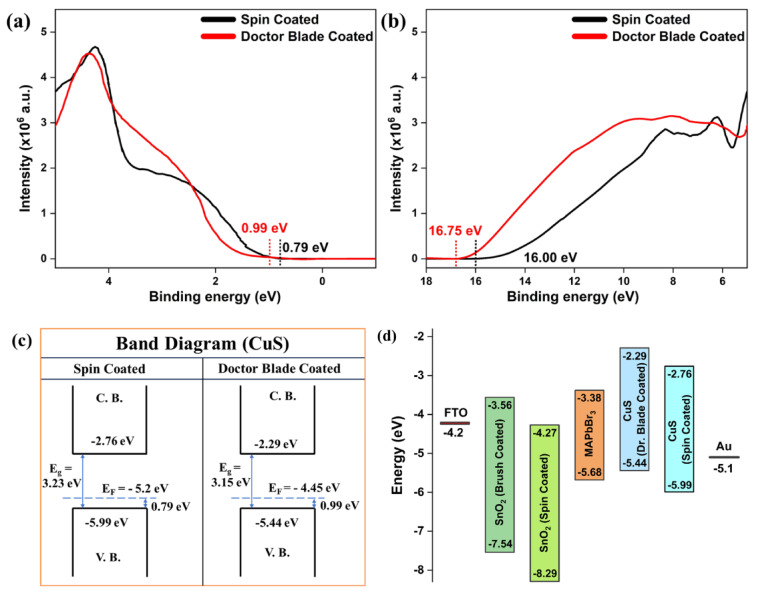
For both Spin- and Doctor Blade-coated thin film samples **(a)** UPS-Valence Band Maximum (VBM) relative to the Fermi level **(b)** UPS-Secondary electron cutoff energy; **(c)** Band energy diagram; **(d)** Band alignment with perovskite.

The energy levels of the valence band for spin-coated and doctor blade-coated films are shown by their distances from the Fermi level (Fig. 4a). The spin-coated film has a valence band maximum (VBM) at 0.79 eV from the Fermi level, while the doctor blade-coated film has its VBM at 0.99 eV, as seen in Fig. 4a. Figure 4b illustrates a cutoff energy of 16.00 eV for the spin-coated film and 16.75 eV for the doctor blade-coated film. This energy is often subtracted from the photon energy in the UPS measurement to ascertain the material’s work function^24^.

The work functions and VBM of spin-coated and doctor blade-coated thin films were determined by ultraviolet photoelectron spectroscopy (UPS), yielding values of −5.2 eV and − 4.45 eV relative to vacuum, respectively. The work functions were calculated by subtracting the UPS HeI radiation energy of 21.2 eV from the high-binding-energy cutoff values: 16.00 eV for the spin-coated film and 16.75 eV for the doctor blade-coated film. This yields − 5.2 eV 0.1 eV relative to vacuum for the spin-coated film, and − 4.45 eV 0.1 eV relative to vacuum for the doctor blade-coated film. The VBM of the spin-coated film was determined to be 0.79 eV below the Fermi level, resulting in a VBM of −5.99 eV relative to the vacuum. Simultaneously, for the doctor blade-coated film, the VBM was ascertained to be 0.99 eV under the Fermi level, equating to a VBM of −5.44 eV relative to the vacuum.

The optical bandgaps were determined to be 3.23 eV for the spin-coated film and 3.15 eV for the doctor blade-coated film (Fig. 2d). Consequently, the conduction band minima (CBM) were calculated as −2.76 eV relative to vacuum for the spin-coated film and − 2.29 eV relative to vacuum for the doctor blade-coated film. The results indicate that the Fermi levels of both spin-coated and doctor blade-coated films are situated closer to the valence band, signifying their p-type characteristics. Figure 4c displays an energy-level diagram, showing the predicted CBMs at −2.76 eV for spin-coated and − 2.29 eV for doctor blade-coated samples, as well as the valence band maxima (VBMs) at −5.99 eV for spin-coated and − 5.44 eV for doctor blade-coated samples.

To facilitate effective charge transfer in perovskite solar cells, the energy levels of the hole transport layer must be precisely matched with those of the perovskite material to minimize energy barriers and mitigate recombination losses. Ideally, the valence band maximum of the hole transport layer should approximate or be somewhat below that of the perovskite. Figure 4d illustrates that the spin-coated material had a VBM of −5.99 eV, much lower than the perovskite’s VBM of −5.68 eV, resulting in a substantial energy offset. The misalignment may have elevated the energy barrier for hole transport from the perovskite to CuS, resulting in greater recombination losses and decreased hole extraction efficiency. Conversely, the doctor blade-coated sample exhibited a VBM of −5.44 eV, much nearer to the perovskite’s VBM, leading to a reduced offset. This configuration proved more advantageous for effective hole extraction, minimizing energy loss and recombination. Optimal band alignment was essential for decreasing energy barriers, improving charge separation and transport efficiency, and minimizing recombination losses when electrons and holes recombined rather than being harvested as electrical current.

## Simulation study

Device-level performance was evaluated via three-dimensional drift–diffusion simulations using the OghmaNano platform. Figure 4d illustrates the energy band alignment of several materials derived from our prior experimental results^4,25^. The FTO/SnO₂ (Brush Coated)/MAPbBr₃/CuS (Spin and Doctor Blade Coated)/Au configuration was chosen for simulation. The CBM of SnO₂ (−3.56 eV) and MAPbBr₃ (−3.38 eV) supports efficient electron transport, while the VBM of CuS (−5.44 eV) aligns well with MAPbBr₃ (−5.68 eV) for effective hole extraction. This alignment minimizes recombination and enhances carrier transport, as illustrated in Fig. 4d, validating the material selection for optimal device performance.

The simulation used the following parameters depicted in Table 2.

**Table 2:** Material parameters (derived from experimental and standard data).

Layer	Band gap (eV)	Electron affinity (eV)	Dielectric constant	Mobility (cm²/Vs)	Thickness (nm)
FTO	3.5	4.4	9.0	20	50
SnO₂ (ETL)	3.9	4.2	10.0	25	200
MAPbBr₃ (Absorber)	2.3	3.9	25.0	3	400
CuS (HTL)	3.1/3.2	4.9	10.5	5	200
Au (Back Electrode)	–	–	–	–	100

**Fig. 5:**
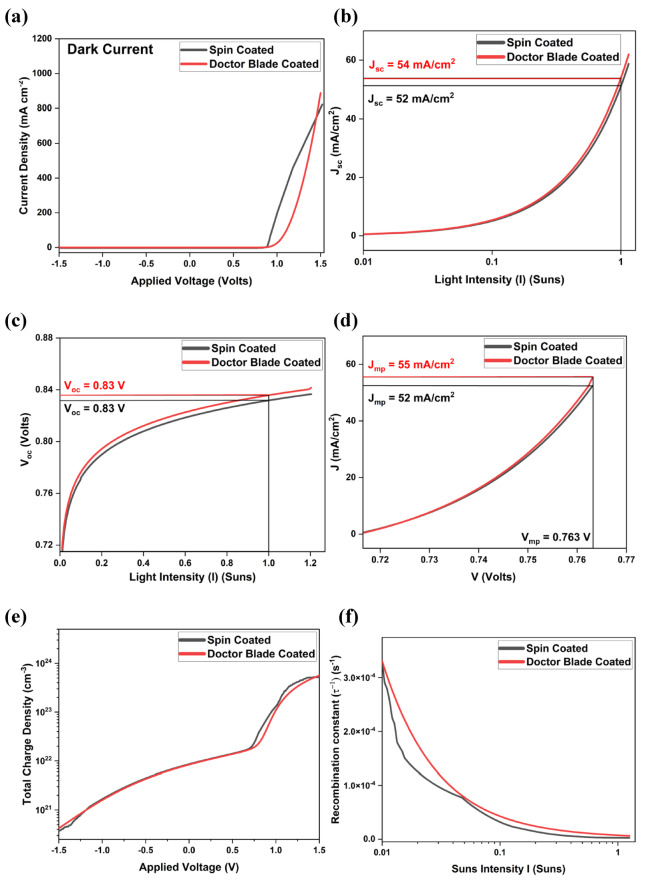
For both Spin- and Doctor Blade-coated thin film samples **(a)** Dark J–V curve showing charge transport; **(b)** Jsc vs. light intensity indicating photocurrent generation; **(c)** Voc vs. light intensity showing recombination behavior; **(d)** J–V at maximum power point; **(e)** Simulated charge density vs. voltage showing charge dynamics;**(f)** Recombination constant vs. light intensity revealing recombination trends.

The simulated J–V characteristics of the FTO/SnO₂/MAPbBr₃/CuS/Au perovskite solar cell (PSC) provide critical insights into its optoelectronic behavior and photovoltaic performance. As shown in Fig. 5a, both spin- and doctor blade-coated devices exhibit negligible reverse leakage and a steep exponential rise in current under forward bias beyond 1 V, confirming strong diode rectification, efficient charge separation, and low recombination—hallmarks of high-quality junctions. The key photovoltaic parameters were extracted under standard AM1.5G illumination (1 SUN). The open-circuit voltage (V_OC_) reaches 0.83 V for both spin- and doctor blade-coated films (Fig. 5c), indicating similarly low non-radiative recombination and effective carrier extraction in both devices. However, the short-circuit current density (J_SC_) is slightly higher for the doctor blade-coated device (54 mA/cm²) compared to the spin-coated device (52 mA/cm²) as shown in Fig. 5b, suggesting improved light absorption and enhanced charge transport efficiency due to better crystallinity and film uniformity in the doctor blade-coated layer.

The J_SC_ variation with illumination intensity in Fig. 5b displays an almost linear relationship for both the spin-coated and doctor blade-coated samples, confirming a highly responsive photodiode behavior and minimal recombination losses under increased light flux. Similarly, Fig. 5c depicts the V_OC_ response concerning light intensity, revealing a logarithmic growth trend that saturates at 0.83 V under full sunlight for both configurations. This trend follows the classical diode equation, where V_OC_ is modulated by the photogenerated current and the associated recombination mechanisms. The consistent rise in V_OC_ at lower intensities also highlights the long carrier lifetimes and negligible shunt losses within the device.

The current density and voltage at the maximum power point (J_mp_ and V_mp_), extracted from Fig. 5d, confirm superior device performance for the doctor blade coated sample with J_mp_ reaching 55 mA/cm² and V_mp_ at 0.76 V. For the spin coated sample, J_mp_ reached 52 mA/cm² with V_mp_ at 0.76 V. These values contribute to an excellent fill factor (FF) of 0.91 for the spin coated sample and 0.93 for doctor blade coated sample, signifying low series resistance and efficient charge collection across the device architecture. Consequently, the overall power conversion efficiency (PCE) is projected to be 39.5% for the spin-coated sample and 41.7% for the doctor blade-coated sample, demonstrating the immense potential of this device configuration for high-efficiency photovoltaic applications.

To further understand the charge carrier dynamics, the variation in total charge density and recombination constants was analyzed for both spin-coated and doctor blade-coated CuS-based devices. These parameters are critical in determining overall device efficiency by directly influencing charge transport, extraction, and recombination behaviors. Figure 5e presents the simulated total charge density as a function of applied voltage. At a reverse bias of −1.5 V, both devices show low charge density, confirming minimal carrier accumulation and the absence of significant leakage or undesirable charge storage. The doctor blade-coated device exhibits a charge density of approximately 10^19^ cm^−3^, slightly higher than the spin-coated counterpart, which records around 8.0 × 10^18^ cm^−3^. As the voltage approaches 0 V, the charge density increases gradually, reaching nearly 1.0 × 10^21^ cm^−3^ for the doctor blade-coated and 8.5 × 10^20^ cm^−3^ for the spin-coated sample. When the forward bias exceeds 0 V, both devices exhibit a sharp exponential rise in charge density; however, the doctor blade-coated device reaches a peak above 10^24^ cm^−3^ at 1.5 V, whereas the spin-coated device saturates around 8.0 × 10^23^ cm^−3^. This pronounced difference suggests greater photogenerated carrier accumulation and possibly more efficient contact-induced injection in the doctor blade-coated film, alongside a slight reduction in carrier extraction efficiency under strong forward bias due to space-charge effects.

Figure 5f illustrates the dependence of the recombination constant (⁻¹) on light intensity for both devices. At low illumination (0.01 Suns), the recombination constant for the doctor blade-coated device peaks at approximately 3.5 × 10^−4^ s^−1^, slightly lower than the spin-coated device, which reaches about 4.2 × 10^−4^ s^−1^, indicating more suppressed trap-assisted Shockley–Read–Hall (SRH) recombination in the former. As light intensity increases to 0.1 Suns, both devices exhibit a sharp decline in recombination constant due to trap filling, with values of approximately 5.0 × 10^−5^ s^−1^ (doctor blade-coated) and 6.2 × 10^−5^ s^−1^ (spin-coated). Under full illumination (1 Sun), the recombination constant further decreases to around 10^−5^ s^−1^ for the doctor blade-coated and 1.3 × 10^−5^ s^−1^ for the spin-coated device, suggesting both have transitioned towards radiative recombination regimes, though the doctor blade-coated sample maintains a lower recombination rate throughout.

These trends confirm that the doctor blade-coated device offers improved carrier generation, higher charge density, and consistently lower recombination losses compared to its spin-coated counterpart, emphasizing its potential for superior photovoltaic performance and enhanced operational stability.

## Conclusion

We have demonstrated that CuS thin films prepared by spin coating and doctor blade coating are effective hole transport layers (HTLs) for perovskite solar cells (PSCs). Both methods yield crystalline, phase-pure films, but they differ in key parameters: spin coating produces smoother, thinner layers with lower defect densities (microstrain ε = 1.31 × 10⁻³; dislocation density δ = 3.83 × 10⁻⁴ nm⁻²), while doctor blade coating yields larger grains (44.9 nm vs. 37.5 nm) and a bandgap of 3.15 eV (vs. 3.23 eV) at the expense of slightly higher microstrain (2.67 × 10⁻³) and defect density (6.89 × 10⁻³ nm⁻²). The PL analysis further confirms that the Doctor Blade Coated film exhibits superior optical quality with lower defects compared to the Spin Coated film. UPS measurements reveal that the doctor blade film’s valence band maximum (–5.44 eV) aligns more closely with MAPbBr₃ (–5.68 eV) than the spin‐coated film (–5.99 eV), facilitating more efficient hole extraction and reduced recombination. Incorporating these parameters into 3D drift–diffusion simulations predicted a higher power conversion efficiency of 41.7% for the doctor blade HTL (vs. 39.5% for spin coating), driven by stronger charge separation and lower recombination losses. These results underscore the scalability and cost-effectiveness of the doctor blade coating method for large-area PSC manufacturing. However, further work is needed to integrate these CuS HTLs into complete device stacks, experimentally validate the simulated performance, and evaluate long-term stability under operational conditions. Such studies will be critical to confirm CuS’s viability for commercial photovoltaic applications.

## Supplementary Information

Below is the link to the electronic supplementary material.

Supplementary Material 1

## Data Availability

All data generated or analyzed during this study are included in this published article. For additional information or data requests, please contact the corresponding author.
