# Supplementary material for: Influence of deposition technique on the structural and optical properties of CuS thin films for hole transport layers
Source: Sci Rep. 2025 Sep 30;15:34020. doi: 10.1038/s41598-025-13226-6 (PMC12485002; doi:10.1038/s41598-025-13226-6)
Supplement: Supplementary file 1 — Supplementary Material 1 [file 41598_2025_13226_MOESM1_ESM.docx]

**Influence of Deposition Technique on the Structural and Optical Properties of CuS Thin Films for Hole Transport Layers**

Tushar A. Limbani^1,a*^, A Mahesh^2,a*^, Shivani R. Bharucha^3,b^

*^a^ C. L. Patel Institute of Studies and Research in Renewable Energy, Charutar Vidya Mandal University, New Vallabh Vidyanagar, Anand, Gujarat - 388121, India.*

*^b^ N. V. Patel College of Pure & Applied Science, Charutar Vidya Mandal University, Vallabh Vidyanagar, Anand, Gujarat- 388120, India.*

Corresponding authors’ email: ^1^email- [tusharlimbani97@gmail.com,](mailto:tusharlimbani97@gmail.com)

^2^email- [maheshiit10@gmail.com](mailto:maheshiit10@gmail.com)


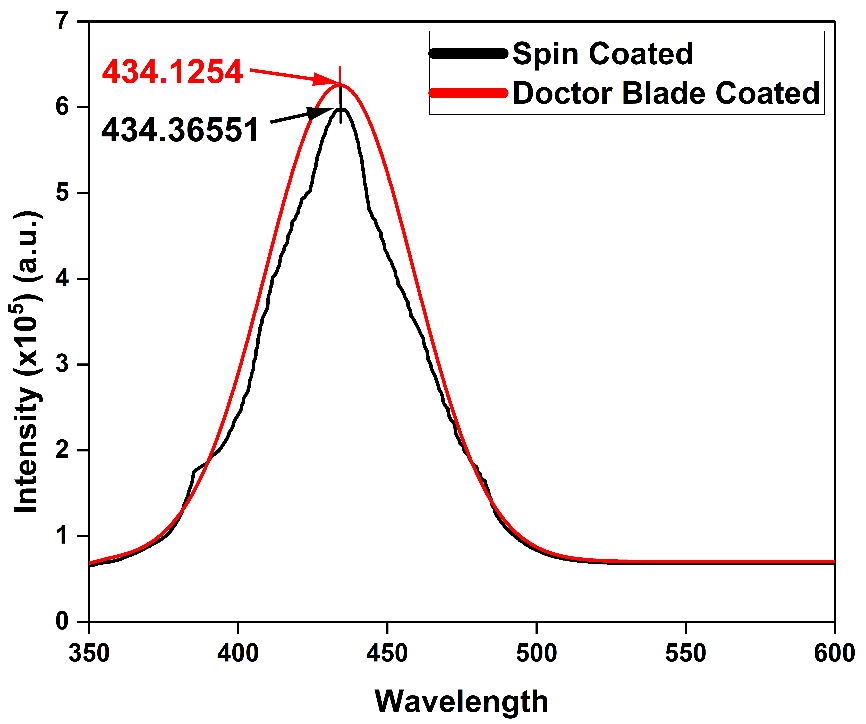


**Figure S1:** PL spectra for both Spin- and Doctor Blade-coated thin film samples.

**Correlation Between PL Observations and Defect Parameters in CuS Thin Films**

Photoluminescence (PL) spectroscopy is a critical technique for evaluating the optical and electronic quality of semiconductor films [1]. In this work, PL data provided key insights into defect-related phenomena such as non-radiative recombination, carrier trapping, and sub-bandgap transitions originating from structural or stoichiometric irregularities [2].

Figure S1 presents the PL spectra of CuS thin films prepared using Spin Coating and Doctor Blade Coating techniques. The emission peaks were observed at 434.37 nm for Spin Coated films and 434.13 nm for Doctor Blade Coated films. The slight redshift (~0.24 nm) in the Doctor Blade film may be attributed to variations in defect-induced states or electronic transitions. Notably, the Doctor Blade film exhibited significantly higher PL intensity, indicating reduced non-radiative recombination, lower defect density, and better crystallinity.

As the CuS layer functions as the Hole Transport Layer (HTL) in our perovskite solar cell architecture, the presence of defects is especially critical. Such defects can introduce deep-level traps that impair hole mobility, increase recombination rates, and ultimately reduce device efficiency [3]. Therefore, PL analysis is not only relevant but integral to refining the simulation parameters for realistic performance modeling. These insights have been incorporated into our simulation models to ensure more accurate device-level predictions.

**References**

1. Li, Q., Anpo, M., You, J., Yan, T. & Wang, X. Photoluminescence (PL) Spectroscopy. in 295–321 (2023). doi:10.1007/978-3-031-07125-6_14.

2. Aalbers, G. J. W. *et al.* Effect of sub-bandgap defects on radiative and non-radiative open-circuit voltage losses in perovskite solar cells. *Nat Commun* 15, 1276 (2024).

3. Sun, R. *et al.* Phase-pure ferroelectric quantum wells with tunable photoluminescence for multi-state optoelectronic applications. *Light Sci Appl* 14, 228 (2025).
